# Glucose metabolic upregulation via phosphorylation of S6 ribosomal protein affects tumor progression in distal cholangiocarcinoma

**DOI:** 10.1186/s12876-023-02815-2

**Published:** 2023-05-16

**Authors:** Atsuro Fujinaga, Teijiro Hirashita, Yuka Hirashita, Kumiko Sakai, Masahiro Kawamura, Takashi Masuda, Yuichi Endo, Masayuki Ohta, Kazunari Murakami, Masafumi Inomata

**Affiliations:** 1 Department of Gastroenterological and Pediatric Surgery, Faculty of Medicine, Oita University, 1-1 Idaigaoka, Hasama-Machi, Oita, 879-5593 Japan; 2 Department of Gastroenterology, Faculty of Medicine, Oita University, Oita, Japan; 3 Molecular Pathology, Faculty of Medicine, Oita University, Oita, Japan; 4 Department of Division of Life Science Research, Faculty of Medicine, Oita University, Oita, Japan

**Keywords:** Distal cholangiocarcinoma, Mammalian target of rapamycin complex 1, Glucose transporter 1, Phosphorylated S6 ribosomal protein, Glycolysis

## Abstract

**Background:**

The prognosis of distal cholangiocarcinoma (dCCA) remains poor; thus, the identification of new therapeutic targets is warranted. Phosphorylated S6 ribosomal protein indicates a mammalian target of rapamycin complex 1 (mTORC1) activity, and mTORC1 plays a central role in controlling cell growth and regulating glucose metabolism. We aimed to clarify the effect of S6 phosphorylation on tumor progression and the glucose metabolic pathway in dCCA.

**Methods:**

Thirty-nine patients with dCCA who underwent curative resection were enrolled in this study. S6 phosphorylation and the expression of GLUT1 were evaluated by immunohistochemistry, and their relationship with clinical factors was investigated. The effect of S6 phosphorylation on glucose metabolism with PF-04691502 treatment, an inhibitor of S6 phosphorylation, was examined in cancer cell lines by Western blotting and metabolomics analysis. Cell proliferation assays were performed with PF-04691502.

**Results:**

S6 phosphorylation and the expression of GLUT1 were significantly higher in patients with an advanced pathological stage. Significant correlations between GLUT1 expression, S6 phosphorylation, and SUV-max of FDG-PET were shown. In addition, cell lines with high S6 phosphorylation levels showed high GLUT1 levels, and the inhibition of S6 phosphorylation reduced the expression of GLUT1 on Western blotting. Metabolic analysis revealed that inhibition of S6 phosphorylation suppressed pathways of glycolysis and the TCA cycle in cell lines, and then, cell proliferation was effectively reduced by PF-04691502.

**Conclusion:**

Upregulation of glucose metabolism via phosphorylation of S6 ribosomal protein appeared to play a role in tumor progression in dCCA. mTORC1 may be a therapeutic target for dCCA.

**Supplementary Information:**

The online version contains supplementary material available at 10.1186/s12876-023-02815-2.

## Background

Currently, cholangiocarcinoma (CCA) is classified as intrahepatic, perihilar and distal, and based on the different anatomical locations, each is characterized by specific epidemiological and clinical features [1, 2]. Distal CCA (dCCA) is highly aggressive: dedifferentiated tumor cells, a strong desmoplastic nature with activation of cell survival and chemoresistance pathways, and high genetic variability, are all factors contributing to its early invasiveness and resistance to therapy [1, 2]. Thus, 5-year survival rates of dCCA remain disappointing, ranging from 20 to 40% [3, 4]. The efficacy of systemic treatment is limited; and therefore, the identification of new therapeutic targets is warranted for patients with dCCA [5].

The phosphatidyl inositol 3-kinase (PI3K)/mammalian target of rapamycin (mTOR) signaling cascade belongs to the critical survival programs that are typically overactivated in dCCA and promote cell survival by inhibiting apoptosis [6]. mTOR is a serine-threonine kinase that functions through two protein complexes, mTOR complex 1 (mTORC1) and mTOR complex 2 [7]. Phosphorylated S6 ribosomal protein (pS6) indicates the activity of mTORC1, and which regulates protein synthesis and cell growth through multiple pathways [8, 9].

Recent studies have shown that mTORC1 is a key regulator of glucose metabolism in cancer [10, 11]. Glucose metabolism is one of the main processes affected by cancer development and metastasis [12]. However, the relation between S6 phosphorylation and glucose metabolism in dCCA is unclear. In this study, we aimed to clarify the effects of S6 phosphorylation on glucose metabolism and tumor progression of dCCA.

## Methods

### Patients

Thirty-nine patients who underwent curative resection for dCCA in the Department of Gastroenterological and Pediatric Surgery, Oita University Faculty of Medicine from 2011 to 2020 were enrolled in this study. Clinicopathological features including patient characteristics, radiological findings, and pathological findings were examined retrospectively. Patient characteristics including age, sex, body mass index, and tumor markers were retrospectively examined, as were the results of blood tests that all patients underwent. Radiological findings including maximum standardized uptake value (SUV-max) of ^18^F-fluorodeoxyglucose positron emission tomography (FDG-PET) /computed tomography and pathological data were obtained from the radiological and pathological reports of our institution. Pathological data included tumor size, tumor type, and T and N status according to the 8^th^ AJCC/UICC TNM classification [13]. Prognosis including the 5-year overall survival (OS) rate was evaluated. S6 phosphorylation and GLUT1 (glucose transporter 1) expression were examined in dCCA tissues by immunohistochemistry. This study was approved by the Ethics Committee of Oita University Faculty of Medicine (#2180).

### Immunohistochemistry

We performed Immunohistochemistry with rabbit polyclonal antibodies against pS6 (S240/244) diluted 1:400 (Cell Signaling Technology, Danvers, MA, USA) and GLUT1 diluted 1:100 (Abcam, Tokyo, Japan). After antigen retrieval, the sections were incubated overnight at 4 °C with antibodies against pS6 and GLUT1 diluted in SignalStain Antibody Diluent (Cell Signaling Technology). After washing with PBS, the sections were incubated with peroxidase-conjugated horse anti- rabbit Ig (ImmPRESS Reagent Kit; Vector Laboratories Inc.) at room temperature for 30 min. ImmPACT DAB Peroxidase Substrate (Vector Laboratories Inc., Newark, CA, USA) was used to detect peroxidase activity.

### Evaluation of immunohistochemistry

**Fig. 1:**
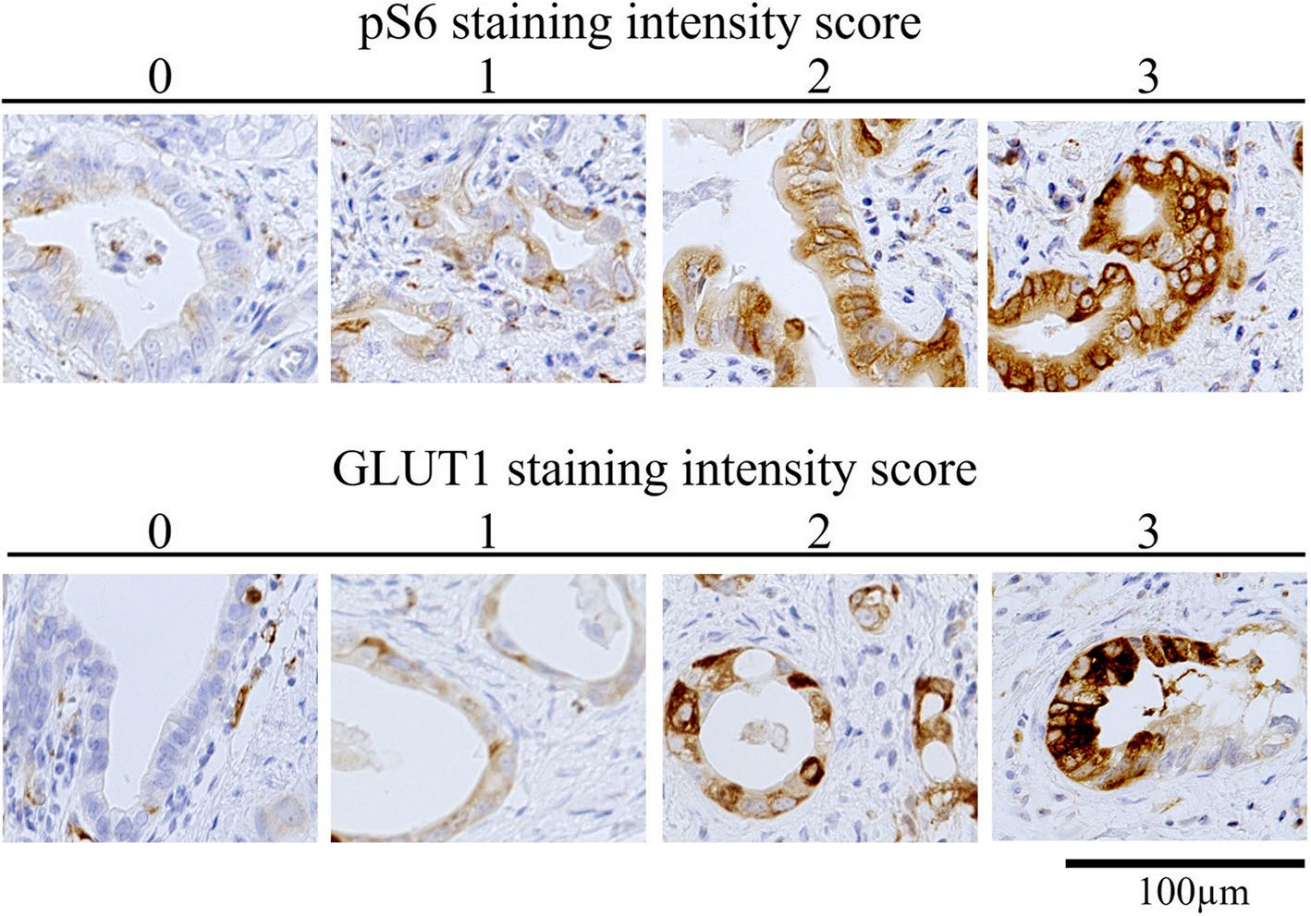
Representative images of immunohistochemistry for scoring of staining intensity for pS6 and GLUT1 in distal cholangiocarcinoma tissues

Two independent observers who had no knowledge of the patients’ outcomes reviewed the slides. Staining intensity was scored as 0, no staining; 1, weak (less than that in fibroblasts in the positive internal control); 2, moderate (as intense as that in positive internal control cells); or 3, strong (more than that in positive internal control cells) (Fig. 1). The staining population was scored based on the ratio of positive cells as follows: 0, 0% of cells positive; 1, 1–25% of cells positive; 2, 25–50% of cells positive; 3, 50–75% of cells positive; and 4, ≥ 75% of cells positive. Scoring was performed in three distinct fields per case, and the three scores were averaged and rounded off to the nearest whole number. In this study, the sum of the intensity and population scores was defined as the pS6 and GLUT1 scores. Patients were divided into two groups (low, < 4 and high, ≥ 4) using the mean values, respectively. Patients were divided into using the mean values, respectively.

### Cell lines

Four human CCA cell lines (MEC, TFK-1, HuCCT1, and TKKK) were obtained from the Japanese Cancer Research Resources Bank (Tokyo, Japan). We cultured all cell lines under the providers' recommended conditions.

### RT-qPCR

Total RNA isolation from cells was performed using an RNeasy Mini Kit (Qiagen, Hilden, Germany). cDNA was synthesized from purified total RNA using a SuperScript VILO cDNA Synthesis Kit (Thermo Fisher Scientific, Waltham, MA, USA). The mRNA expression levels were assessed with LightCycler 480 SYBR Green I Master (Roche Diagnostics, Mannheim, Germany). Primer pairs were as follows: GLUT1 (forward: 5′-GTCAACACGGCCTTCACTG-3′; reverse: 5′-GGTCATGAGTATGGCACAACC-3′), β-actin (forward: 5′-AGAGCAAGAGAGGCATCCTC-3′; reverse: 5′-CTCAAACATGATCTGGGTCA-3′). mRNA expression levels were normalized to that of β-actin.

### Western blotting

Western blotting was performed as previously described [14]. The separated proteins from cells were transferred to a polyvinylidene difluoride membrane (Millipore). The membranes were incubated with primary antibodies against pS6 (S240/244) diluted 1:2000 (Cell Signaling Technology), tS6 diluted 1:2000 (Cell Signaling Technology), GLUT1 diluted 1:1000 (Abcam), HK-1 diluted 1:1000 (Abcam), cleaved caspase 3 diluted 1:1000 (Cell Signaling Technology)and GAPDH diluted 1:1000 (Santa Cruz Biotechnology, Santa Cruz, CA, USA).

### Cell proliferation assay

Cell proliferation assays were performed as previously described [14]. Cells were seeded at densities of 4 × 103 cells/well in 96-well plates. The cells were incubated for 24 h and then treated with DMSO or the serial dilutions (0.01, 0.1, 1.0, 10, and 100 µmol/L) of PF-04691502 (Selleck Chemicals, Houston, TX, USA), an inhibitor of S6 phosphorylation, for 72 h. The growth-inhibitory effect of the treatment was analyzed by CellTiter 96 AQueous One Solution Cell Proliferation Assay (Promega) in quadruplicate and expressed as the GI50 value, which was calculated using the linear relationship between the percentage inhibition and log concentration.

### Invasion and migration assay

The invasion assay was performed using 24-well Transwell culture chambers (Corning). The lower and upper surfaces of filters with an 8.0-μm pore size were coated with 1 μg of fibronectin and 5 μg of Matrigel (only in the invasion assay), respectively. In total, 1 × 105 CCA cells in 100 μL of medium were added to the upper compartment of the chamber, and 600 μL of medium with DMSO (Mock) or PF-04691502 (0.1 µmol/L) was added to the lower chamber. After incubation for 24 h, the filter was fixed with 30% methanol and stained with 0.5% crystal violet in 20% ethanol. Then, the non-invading cells on the upper surface of the filter were removed using a cotton swab. Subsequently, the filter was excised carefully from the chamber with a scalpel and placed in the 96-well plate. Then, 100 μL of 30% acetic acid was added to each well to decolorize the filter. The number of stained cells on the filter was estimated by measuring the absorbance of the supernatant using a SPECTRAFluor plate reader (Tecan, Männedorf, Switzerland).

### Metabolomics analysis

Two experimental groups were prepared: untreated control TFK-1 (*n* = 5) and TFK-1 cultured with 1 µM PF-04691502 (nI = 5) for 24 h. Targeted metabolomics analysis was performed using gas chromatography-tandem mass spectrometry (GC–MS/MS). For the analysis, we used a GCMS-TQ8040 system (Shimazu Corporation, Kyoto, Japan) equipped with a DB-5 capillary column (30 × 0.25-mm inner diameter, film thickness 1 µm; Agilent). Control settings and running conditions of the system were as described previously [15]. The MRM analysis method included in the Smart Metabolites Database version 3 (Shimadzu Corporation) was used to detect metabolites, and samples were analyzed based on the 469 metabolites database. The internal standard used to evaluate the validity of the analytical result was 2-isopropylmalic acid. Metabolite peak signals were automatically identified according to precursor, product ions, and specific retention time.

### Multivariate analysis of metabolomics data

The metabolomics dataset with peak areas of 469 metabolite signals was imported into SIMCA software version 13.0.3.0 (Sartorius Stedim Biotech, Göttingen, Germany). To visualize the differences between the metabolomics datasets and extract the significant metabolites, we performed orthogonal projections to latent structures discriminant analysis (OPLS-DA) with Pareto scaling. In the S-plot, compounds with absolute values of P(corr) [1] > 0.7 were considered to be significant metabolites. For pathway analysis, we used MetaboAnalyst version 5.0 [16] and the Small Molecule Pathway Database (www.metaboanalyst.ca) [17].

### Statistical analysis

All variables are expressed as the mean ± standard deviation for continuous data. Differences between the variables were compared using Fisher’s exact test or the Mann–Whitney U test. Correlation between the continuous variables was investigated using Spearman’s rank correlation coefficient. Survival curves were estimated using the Kaplan–Meier method, and the log-rank test was used to detect differences between curves. *P* values of < 0.05 were considered to indicate statistical significance. All statistical analyses were performed with JMP software version 14.2 for Windows (SAS Institute, Cary, NC, USA).

## Results

### Clinicopathological features

The study population included 39 patients (female, *n* = 8; male, *n* = 31; mean age, 73.8 ± 6.5 years) (Table 1). The serum levels of CEA (carcinoembryonic antigen) and CA19-9 were 3.9 ± 2.9 ng/mL and 659.0 ± 2284.9 U/mL, respectively. The mean SUV-max of FDG-PET was 7.5 ± 5.2, and the mean tumor diameter was 35.8 ± 16.4 mm. T Stages were I in 2, II in 18, III in 14, and IV in 5 patients. Lymph node metastasis was found in 14 patients, and lymphatic vessel invasion and venous invasion were found in 21 patients each. UICC Stages were I in 2, IIA in 13, IIB in 16, IIIA in 3, IIIB in 5, and IV in 0 patients. The mean GLUT1 score and pS6 score were 4.0 ± 1.5 and 3.9 ± 1.4, respectively. The 5-year OS rate was 54.2%.

**Table 1:** Patient characteristics

	*n* = 39
Age (y)	73.8 ± 6.5
Sex (male/female)	31/8
Body mass index (kg/m^2^)	22.2 ± 3.4
CEA (ng/mL)	3.9 ± 2.9
CA19-9 (U/mL)	659.0 ± 2284.9
SUV-max of FDG-PET	7.5 ± 5.2
Tumor size (mm)	35.8 ± 16.4
Tumor type (Papillary/Nodular/Flat)	2/25/12
T Stage (1/2/3/4)	2/18/14/5
Nodal status (N0/N1/N2)	25/11/3
Lymphatic invasion (-/ +)	18/21
Vascular invasion (-/ +)	18/21
Nerve invasion (-/ +)	9/30
UICC Stage (I/IIA/IIB/IIIA/IIIB/IV)	2/13/16/3/5/0
pS6 score	3.9 ± 1.4
GLUT1 score	4.0 ± 1.5

*CEA* carcinoembryonic antigen, *SUV-max m*aximum standardized uptake value, *UICC* Union for International Cancer Control, *pS6* phosphorylated S6 ribosomal protein, *GLUT1* glucose transporter 1

### Relationship between pS6 or GLUT1 and clinicopathological features

According to the immunohistochemical expression of pS6, 17 patients showed high expression levels, and 22 showed low expression levels, and the CA19-9 level, SUV-max of FDG-PET, T stage, and UICC Stage were significantly higher in the pS6-high group (Table 2). Regarding GLUT1, 17 patients showed high expression levels, and 22 showed low expression levels, and the CA19-9 level, SUV-max of FDG-PET, T stage, nodal status, lymphatic invasion, and UICC Stage were significantly higher in the GLUT1-high group (Table 2). The 5-year OS rates were 65.6% in the pS6-low group and 37.1% in the pS6-high group (*P* = 0.266) and 63.1% in the GLUT1-low group and 40.5% in the GLUT1-high group (*P* = 0.163).

**Table 2:** Relationship between S6 phosphorylation or GLUT1 and patient characteristics

	pS6 < 4 (*n* = 22)	pS6 ≥ 4 (*n* = 17)	*P* value
Age (y)	74.1 ± 7.4	73.5 ± 5.2	0.792
Sex (male/female)	18/4	13/4	0.683
Body mass index (kg/m^2^)	22.3 ± 3.1	22.2 ± 3.9	0.882
CEA (ng/mL)	3.3 ± 1.9	4.7 ± 3.7	0.144
CA19-9 (U/mL)	23.4 ± 21.7	1481.5 ± 3335.3	0.047*
SUV max of FDG-PET	4.8 ± 0.7	11.2 ± 6.4	0.002*
Tumor size (mm)	34.1 ± 17.0	38.1 ± 15.8	0.456
Tumor type (Papillary/Nodular/Flat)	2/13/7	0/12/5	0.080
T Stage (≤ 2/3 ≤)	16/6	4/13	0.002*
Nodal status (N0/N1/N2)	16/4/2	9/7/1	0.285
Lymphatic invasion (-/ +)	13/9	5/12	0.063
Vascular invasion (-/ +)	13/9	5/12	0.063
Nerve invasion (-/ +)	6/16	3/14	0.475
UICC Stage (≤ IIA/IIB ≤)	13/9	2/15	0.003*
	GLUT-1 < 4 (*n* = 22)	GLUT-1 ≥ 4 (*n* = 17)	*P* value
Age (y)	73.9 ± 7.3	73.7 ± 5.3	0.946
Sex (male/female)	18/4	13/4	0.683
Body mass index (kg/m^2^)	22.1 ± 3.1	22.4 ± 3.8	0.748
CEA (ng/mL)	3.3 ± 1.9	4.7 ± 3.7	0.118
CA19-9 (U/mL)	24.3 ± 21.8	1480.4 ± 3335.8	0.047*
SUV max of FDG-PET	4.8 ± 1.3	10.6 ± 1.4	0.007*
Tumor size (mm)	35.5 ± 19.3	36.2 ± 4.0	0.899
Tumor type (Papillary/Nodular/Flat)	2/12/8	0/13/4	0.379
T Stage (≤ 2/3 ≤)	15/7	5/12	0.025*
Nodal status (N0/N1/N2)	18/2/2	7/9/1	0.009*
Lymphatic invasion (-/ +)	14/8	4/13	0.023*
Vascular invasion (-/ +)	13/9	5/12	0.063
Nerve invasion (-/ +)	7/15	2/15	0.251
UICC Stage (≤ IIA/IIB ≤)	13/9	2/15	0.003*

*pS6* phosphorylated S6 ribosomal protein*, CEA* carcinoembryonic antigen*, SUV-max* maximum standardized uptake value, *UICC* Union for International Cancer Control, *GLUT1* glucose transporter 1

^*^ *P* < 0.05

### Correlation between GLUT1, pS6, and the SUV-max of FDG-PET

**Fig. 2:**
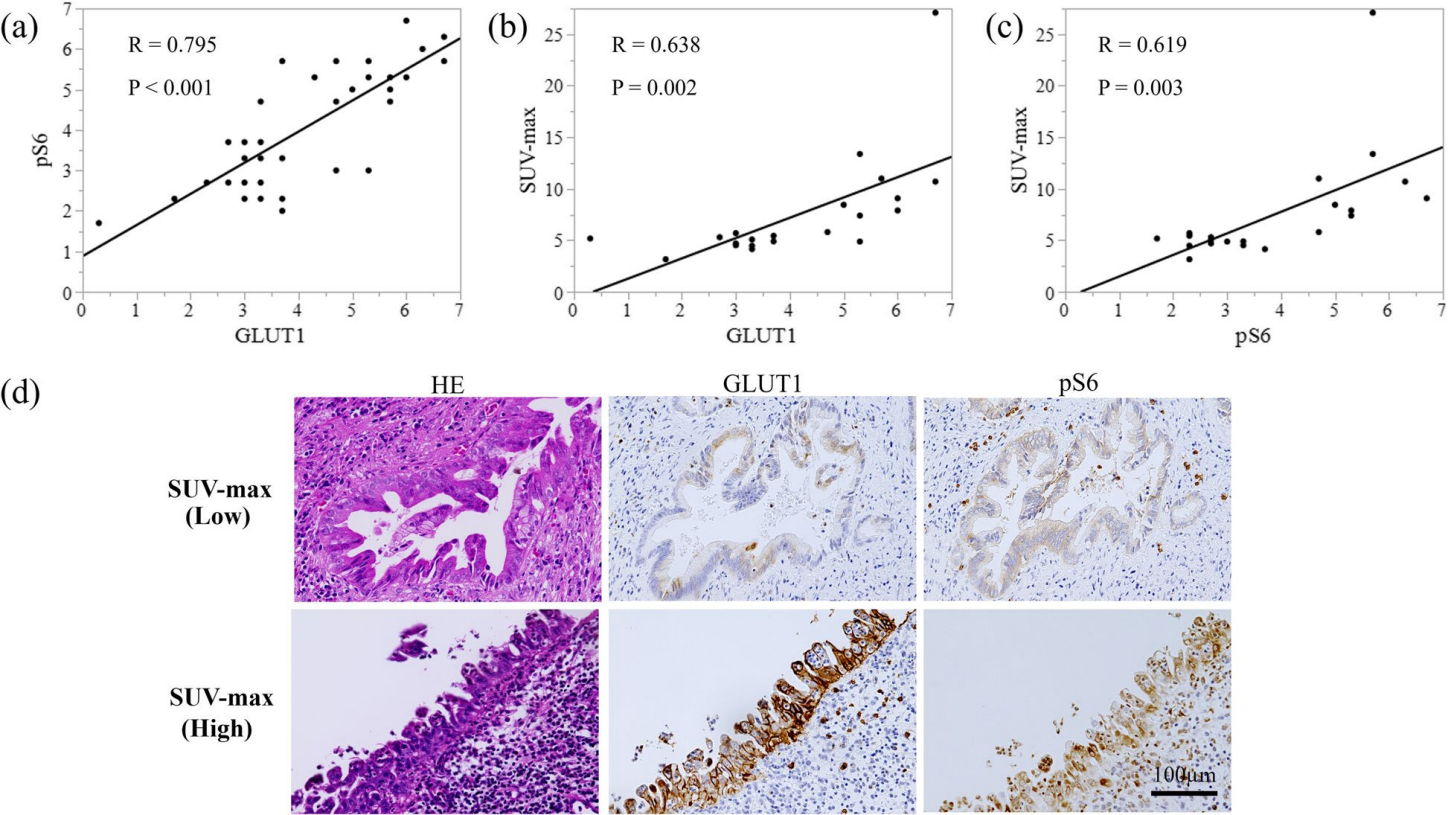
**a**-**c** Scatter plots showing the relationship between GLUT1 and pS6, GLUT1 and SUV-max of FDG-PET, and pS6 and SUV-max of FDG-PET. **d** Representative images of immunohistochemistry using antibodies against GLUT1 and pS6 in distal cholangiocarcinoma tissues

We further investigated to confirm the relationship between GLUT1 expression, pS6 level, and SUV-max in dCCA. Significant correlations were found between GLUT1 and pS6 (*R* = 0.795, *P* < 0.001), GLUT1 and SUV-max (*R* = 0.638, *P* = 0.002), and pS6 and SUV-max (*R* = 0.619, *P* = 0.003) (Fig. 2).

### Relationship between S6 phosphorylation and glucose metabolism and distal cholangiocarcinoma cell lines

**Fig. 3:**
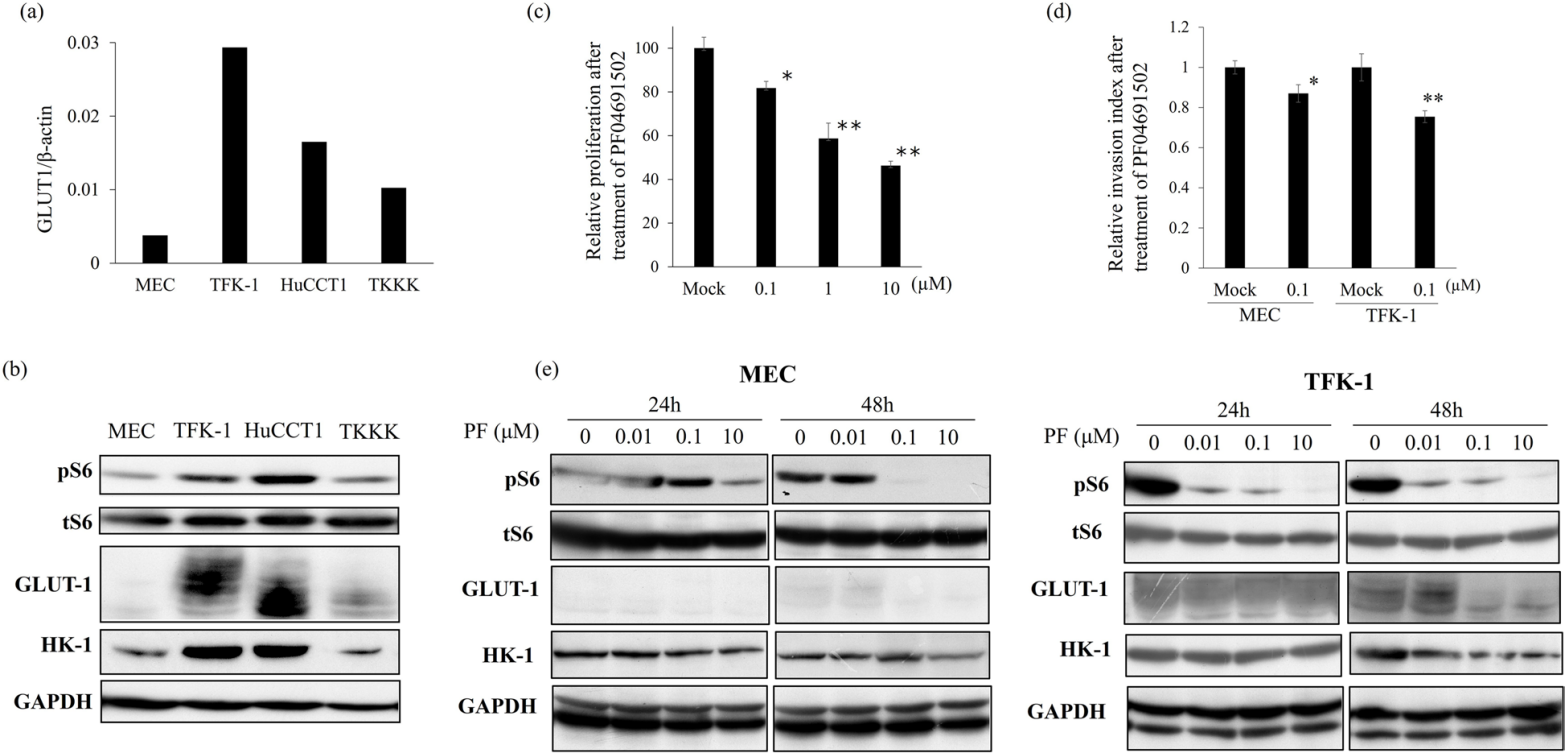
**a** mRNA expression of GLUT1 in 4 cholangiocarcinoma cell lines. **b** Western blotting to investigate the expression of GLUT1 and S6 phosphorylation in 4 cholangiocarcinoma cell lines. **c** The inhibitory effect of PF-04691502 treatment on cell proliferation in TFK-1 cells. **d** The inhibitory effect of PF-04691502 (0.1 µmol/L) treatment on invasiveness in TFK-1 and MEC cells. **e** Western blotting to investigate the effect of S6 phosphorylation on GLUT1 expression under PF-04691502 treatment. * vs control, *P* < 0.05, ** vs control, *P* < 0.001

The mRNA expression of GLUT1 was examined in four cell lines by RT-qPCR and was found to be high in TFK-1 and HuCCT1 cells (Fig. 3a). The protein expression of GLUT1 and HK-1, and S6 phosphorylation were also examined in the four cell lines by Western blotting. TFK-1 and HuCCT1 cells showed high GLUT1 and HK-1 expression levels. The S6 phosphorylation level was also high in these same two cell lines. In contrast, MEC and TKKK cells showed low S6 phosphorylation levels and low GLUT1 and HK-1 expression levels (Fig. 3b). We also investigated the effect of the inhibition of S6 phosphorylation using PF-04691502 treatment for TFK-1 with high S6 phosphorylation and MEC with low S6 phosphorylation. TFK-1 was effectively reduced by PF-04691502 treatment in the cell proliferation assay (GI50 = 2.40 µmol/L) (Fig. 3c, Supplementary Fig. 1a) and invasion assay (Fig. 3d). However, there was no significant change in migration ability after treatment with PF-04691502 (Supplementary Fig. 1b). We also investigated the level of cleaved caspase3 to determine whether PF-04691502 treatment induces apoptosis in TFK-1 and MEC cells. As shown in Supplementary Fig. 1b, inhibition of mTORC1 did not induce apoptosis in either cell line. The level of S6 phosphorylation was clearly suppressed in TFK-1 by using PF-04691502. The GLUT1 and HK-1 expressions were also clearly suppressed in TFK-1 after 48 h treatment (Fig. 3d, Supplementary Fig. 1d). The levels of S6 phosphorylation and expression of GLUT1 were slightly suppressed in MEC and TKKK cells by using PF-04691502 (Fig. 3d, Supplementary Fig. 1d).

### Metabolomics analysis of TFK-1 cell line under PF-4691502 treatment

**Fig. 4:**
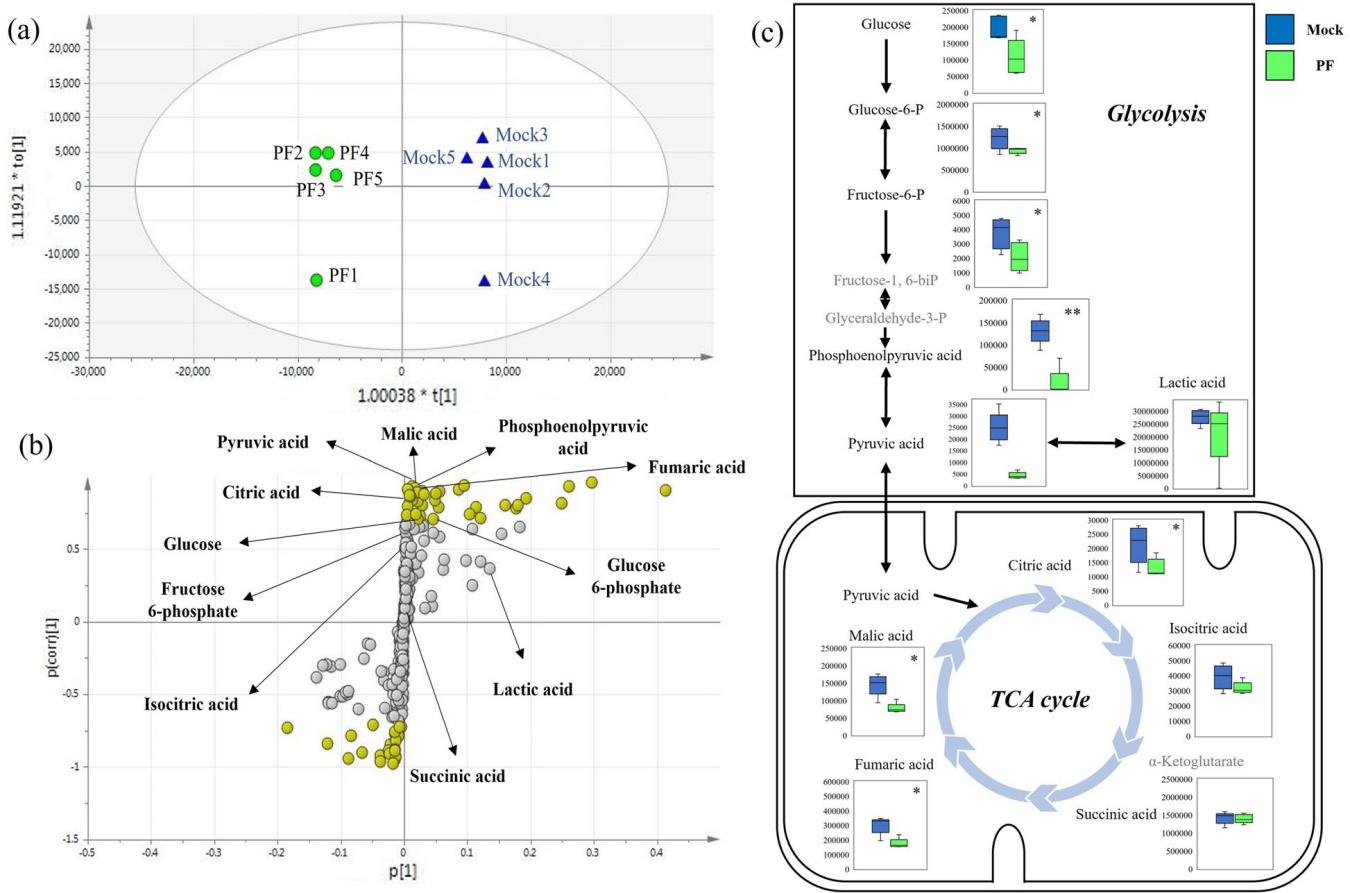
Metabolomics analysis of TFK-1 cell line under PF-04691502 treatment. **a** OPLS-DA score scatter plot of the TFK-1 cell line under mock and PF-04691502 treatment. **b** Corresponding S-plot of the TFK-1 cell line under mock and PF-04691502 treatment. The metabolites with absolute value of P(corr)[1] > 0.7 are highlighted with yellow circles. The symbols of P[1] and P(corr)[1] are *P* values, which represent the contribution to the relative quantities of each metabolite and the difference between groups, respectively. Metabolomics related to pathways of glycolysis and the TCA cycle are shown. **c** Metabolomics related to pathways of glycolysis and the TCA cycle. Signal intensities of each metabolite measured by GC–MS/MS are shown in the bow-whisker plots. * vs control, *P* < 0.05, ** vs control, *P* < 0.001

We next investigated the change of metabolites after PF-04691502 treatment in TFK-1. Fifty-eight of the 469 metabolites were significantly extracted by GC–MS/MS. The metabolic differences between the two groups with and without PF-04691502 treatment were investigated using OPLS-DA, and differential metabolic patterns were seen by the two plots clearly separated on the score scatter plot (Fig. 4a). Subsequently, an S-plot was created to select important metabolites with which to distinguish the two groups (Fig. 4b). Fifty-five metabolites with absolute values of P(corr)[1] > 0.7, marked with yellow circles in Fig. 4b, were selected as the main discriminating compounds (Table 3). To identify the metabolic pathways in which the 55 selected metabolites were involved in, pathway analyses were performed. Pathway analysis showed that the pathways of glycolysis and the TCA (tricarboxylic acid cycle) cycle were significantly reduced in the group treated with PF (*P* = 0.010 and *P* = 0.003), respectively. Metabolomics related to pathways of glycolysis and the TCA cycle are shown in Fig. 4b and c.

**Table 3:** Metabolites selected by S-plot of the TFK-1 cell line under PF-04691502 treatment

No	Metabolite	P[1]^a^	P(corr)[1]^a^	No	Metabolite	P[1]^a^	P(corr)[1]^a^
1	Putrescine	0.296	0.958	29	Homocysteine	0.022	0.722
2	Uridine monophosphate	0.095	0.934	30	Ornithine	0.120	0.715
3	Pyruvic acid	0.013	0.933	31	Glucose 6-phosphate	0.047	0.707
4	Taurine	0.261	0.933	32	Asparagine	0.024	0.707
5	Cytosine	0.017	0.918	33	Glucose	0.022	0.705
6	2'-Deoxyuridine	0.006	0.911	34	Niacinamide	-0.049	-0.714
7	4-Aminobutyric acid	0.087	0.908	35	Tryptophan	-0.008	-0.723
8	Guanine	0.008	0.902	36	Methylsuccinic acid	-0.005	-0.729
9	5-Aminovaleric acid	0.056	0.900	37	Galactose	-0.185	-0.730
10	Phosphoenolpyruvic acid	0.029	0.895	38	O-Phospho-Serine	-0.015	-0.763
11	N-Acetylaspartic acid	0.020	0.893	39	3-Hydroxyisovaleric acid	-0.015	-0.770
12	Adenine	0.050	0.882	40	3-Hydroxy-3-methylglutaric acid	-0.009	-0.788
13	Dopamine	0.031	0.874	41	Stearic acid	-0.083	-0.789
14	Cystathionine	0.010	0.861	42	Lyxose	-0.013	-0.821
15	Creatinine	0.050	0.835	43	3-Aminopropanoic acid	-0.120	-0.844
16	2-Phosphoglyceric acid	0.012	0.833	44	Decanoic acid	-0.022	-0.847
17	Malic acid	0.021	0.829	45	Octanoic acid	-0.015	-0.871
18	Glutamine	0.250	0.814	46	Arabinose	-0.014	-0.883
19	Fumaric acid	0.028	0.802	47	Serotonin	-0.014	-0.887
20	Spermidine	0.160	0.798	48	Lauric acid	-0.025	-0.888
21	Histidine	0.180	0.798	49	Nonanoic acid	-0.066	-0.903
22	Citric acid	0.008	0.798	50	Ribulose	-0.023	-0.911
23	Dihydroorotic acid	0.006	0.792	51	Benzoic acid	-0.038	-0.920
24	2-Aminoethanol	0.055	0.790	52	N-Acetyltyrosine	-0.014	-0.929
25	Fructose 1-phosphate	0.113	0.789	53	Glycine	-0.089	-0.944
26	Hydroxylamine	0.178	0.783	54	Dimethylglycine	-0.014	-0.949
27	Spermine	0.019	0.742	55	Nicotinic acid	-0.038	-0.964
28	Saccharopine	0.005	0.735				

^a^The data in the columns labelled “P[1]” and “P(corr)[1]” are *P* values representing the contribution to the relative quantities of each metabolite and the difference between groups, respectively

## Discussion

dCCA is a highly aggressive type of malignancy. As patients in the early stages of dCCA are frequently asymptomatic, most patients receive a diagnosis at an advanced or locally unresectable stage of the disease, ending in a poor prognosis with limited systemic treatment options [18]. Combination platinum-gemcitabine chemotherapy is an active first-line treatment regimen [19] but it is associated with low rates of radiologic response and short time to tumor progression. The identification of new therapeutic targets is warranted to develop effective new treatments for patients with dCCA. The collective evidence of genetic studies in CCA has suggested that mTOR plays a central and critical role in invasive CCA, and therefore, targeting the mTOR pathway with mTOR inhibitors could be envisioned as a novel treatment [20]. The mechanism involves the regulation of glucose metabolism, which is important in cancer, by mTORC1 [10–12]. However, the role of mTORC1 and the relation between glucose metabolism and tumor progression in dCCA are unclear. The present study has revealed some new findings. S6 phosphorylation and the expression of GLUT1 were significantly higher in dCCA patients with an advanced pathological stage. In addition, a significant correlation was observed between S6 phosphorylation and the expression of GLUT1 in both dCCA patients and cell lines. Furthermore, mTORC1 inhibition induced suppression of the expression of GLUT1 and led to suppression of the glycolysis pathways in dCCA cell lines, which implicate the role of mTORC1-driven glucose metabolism in dCCA.

The most common and one of the first-identified biochemical features of cancer cells is glucose metabolism. Glucose is known as a primary energy and carbon source for the cells, providing not only energy in the form of ATP but also metabolites for several anabolic pathways [21]. Cancer cells characteristically show increased glucose uptake and glycolytic rate, leading to activation of the TCA cycle. This process, known as the Warburg effect, is aerobic glycolysis as it occurs even in the presence of adequate oxygen to maintain mitochondrial respiration [22]. Interestingly, the upregulation of glycolysis has been reported in CCA patients [23], and glucose transporters such as GLUT1 and HK-1 help in this uptake of glucose [24]. FDG-PET is an imaging tool that estimates the metabolic status of glucose in many tumors and shows promise for detecting and localizing biliary cancer [25]. FDG-PET is frequently used for initial staging, evaluation of response to treatment, and prognosis of biliary cancer [26, 27]. Yoon et al. reported that GLUT1 expression of tumor tissue was correlated with FDG uptake in dCCA [28]. In the present study, similar to previous reports [28], the expression level of GLUT1 was correlated with SUV-max of FDG-PET. In addition, the GLUT1 level was significantly higher in patients with advanced dCCA. These results suggested that upregulation of glucose metabolism is important in dCCA progression.

With the improvement of cancer genome profiling technology, a variety of molecular alterations involving both oncogenes and tumor suppressor genes have been described in CCA [29], and most of the genetic alternations in dCCA involve PI3K/mTOR [6]. The serine/threonine kinase mTOR, a member of the family of protein kinases called PI3K-related kinases, integrates intracellular and extracellular signal transduction leading to regulation of various cellular functions such as cell cycle progression, cell metabolism, cell proliferation, and survival. mTORC1 regulates protein synthesis and cell growth through multiple pathways, including 2 well-characterized downstream molecules, 4E-BP1 and ribosomal protein 70S6 kinase (70S6K) [7]. Upon activation, mTORC1 phosphorylates 4E-BP1 (p4EBP1) and 70S6K (p70S6K), which subsequently phosphorylate ribosomal protein S6 [8, 9]. Expression of pS6, p70S6K, and p4EBP1 are well-established readouts of mTORC1 activation. A previous report showed that gene expression profiling of invasive CCA has indicated upregulation of downstream mediators in the mTOR pathway [20]. Immunohistochemistry of the PI3K/mTOR pathway has suggested that up to 50% of CCA may harbor activation of this pathway, which was associated with poor prognosis [30]. In the present study, S6 phosphorylation, which indicates the activity of mTORC1, as examined by immunohistochemistry was significantly higher in dCCA patients with an advanced T stage and pathologic stage. In other words, the mTOR pathway including mTORC1 was activated in advanced CCA.

As previously described, mTORC1 is associated with a variety of factors related to cancer progression, including its role as including its role as a key regulator of glucose metabolism [10, 11]. The present study additionally showed that activated mTORC1 can lead to upregulation of glucose metabolism in dCCA. We found significant correlations between GLUT1 expression, S6 phosphorylation, and glucose uptake (FDG SUV-max) in the dCCA patients. Furthermore, even in vitro, two of the four cell lines with high GLUT1 expression showed a high level of S6 phosphorylation. We also investigated the effect of the decrease in mTORC1 activity using PF-04691502 treatment, which inhibits the PI3K/mTOR pathway. The high levels of S6 phosphorylation in TFK-1 were markedly reduced after treatment with PF-04691502. Metabolomic analysis showed a significant decrease in metabolomics related to glycolysis and the TCA cycle in TFK-1 after treatment with PF-0491502, and pathway analysis also showed a significant reduction in glycolysis and the TCA cycle. Therefore, in dCCA, activation of mTORC1 may also have a potential pivotal role in the regulation of glucose metabolism. Conversely, it is noteworthy that FDG-PET may clinically reflect the activity of mTORC1. In an in vitro experiment, PF-04691502 targeting mTORC1 significantly inhibited cell proliferation and invasiveness, indicating that mTORC1 is a potential therapeutic target.

According to the ABC-02 trial and FUGA-BT study, the combination therapy of gemcitabine plus cisplatin or TS-1 can be considered a standard treatment in patients with advanced CCA [19, 31]. Regarding the mTOR/PI3K pathway, mTOR inhibitors are approved for the treatment of renal cell, neuroendocrine, and hormone-positive/HER-2-negative advanced breast cancer [32–34]. To date, however, in CCA, to date, regimens using molecularly targeted agents including, PF-04691502 have not shown an additional effect over that of standard therapy. One recent study showed that gemcitabine-resistance is associated with increased glucose uptake in cancer cells [35], and as shown in Supplementary Fig. 2, TFK-1 cells, which have a high level of pS6, showed resistance to gemcitabine. Interestingly, PF-04691502 combination therapy with gemcitabine showed a significant growth-inhibitory effect in TFK-1 cells. Although further investigation including metabolomics analysis after gemcitabine and PF-04691502 treatment is necessary, it is possible that mTORC1 inhibition combined with gemcitabine would enhance the growth-inhibitory effect on gemcitabine-resistant CCA cells by reducing glucose metabolism. Clinical trials for some carcinomas using PF-04691502 were reported [36, 37], but its efficacy has not been proved and a high rate of adverse events has been reported. Several studies in different cancer types have already shown that inhibition by targeted therapy only for mTORC1 signaling is strongly associated with primary and acquired resistance to such treatment [38, 39], and this inhibition leads to the release of negative feedback and activation of Akt via PI3K, which is thought to attenuate the antitumor effect. Therefore, it is considered that mTORC1 inhibition seems to be an effective therapeutic strategy in combination with other targeted agents even though the efficacy is limited as a single-use treatment [38, 39].

This study has some limitations. First, this is a retrospective study with a small sample size. Thus, a larger prospective study will be necessary. Second, the effects of an mTORC1 inhibitor have only been investigated in in vitro experiments using cell lines. Further studies are needed to clarify the relation between S6 phosphorylation and glucose metabolism and cancer progression in vivo using experimental animals. Third, HuCCT1 and TKKK cell lines were derived from intrahepatic CCA [40–43]. Our results showed HuCCT1 cells to have high levels of mTORC1 activity (Fig. 3b) and to be highly sensitive to PF-04691502 (Supplementary Fig. 1a). Although we performed immunohistochemical staining only in specimens from patients with dCCA, a relationship between the mTORC1 signal and glucose metabolism was detected in intrahepatic CCA cell lines. Therefore, further study that includes intrahepatic CCA and perihilar CCA will be required.

## Conclusion

In conclusion, S6 phosphorylation may play an important role in tumor progression connected with the upregulation of glucose metabolism in dCCA. mTORC1 may be one of the therapeutic targets for dCCA, and novel combination therapy with mTORC1 inhibitors may potentially be warranted.

## Supplementary Information

**Additional file 1:** **Supplementary Fig. 1.**The inhibitory effect of PF-04691502 treatment on cell proliferation in four CCA cell lines.The inhibitory effect of PF-04691502treatment on migration ability in TFK-1 and MEC cells.Western blotting to investigate the level of cleaved caspase3 under PF-04691502 treatment in TFK-1 and MEC cells. * vs control, *P* < 0.05, ** vs control, *P* < 0.001.

**Additional file 2:** **Supplementary Fig. 2.** The inhibitory effect of cisplatin/gemcitabinecombined with PF-04691502in TFK-1 and MEC cells. * vs control, *P* < 0.05, ** vs control, *P* < 0.001.

**Additional file 3.**

## Data Availability

The data that support the findings of this study are available on request to the corresponding author.

## Abbreviations

mTORC1: Mammalian target of rapamycin complex 1;
dCCA: Distal cholangiocarcinoma
CCA: Cholangiocarcinoma
PI3K: Phosphatidyl inositol 3-kinase
mTOR: Mammalian target of rapamycin
pS6: Phosphorylated S6 ribosomal protein
SUV-max: Maximum standardized uptake value
FDG-PET: ^18^F-fluorodeoxyglucose positron emission tomography/computed tomography
OS: Overall survival
GLUT1: Glucose transporter 1
OPLS-DA: Orthogonal projections to latent structures discriminant analysis
70S6K: Ribosomal protein 70S6 kinase
p4EBP1: Phosphorylates 4E-BP1
p70S6K: Phosphorylates 70S6K
