# Supplementary figures and images for: Glucose metabolic upregulation via phosphorylation of S6 ribosomal protein affects tumor progression in distal cholangiocarcinoma
Source: BMC Gastroenterol. 2023 May 16;23:157. doi: 10.1186/s12876-023-02815-2 (PMC10190040; doi:10.1186/s12876-023-02815-2)

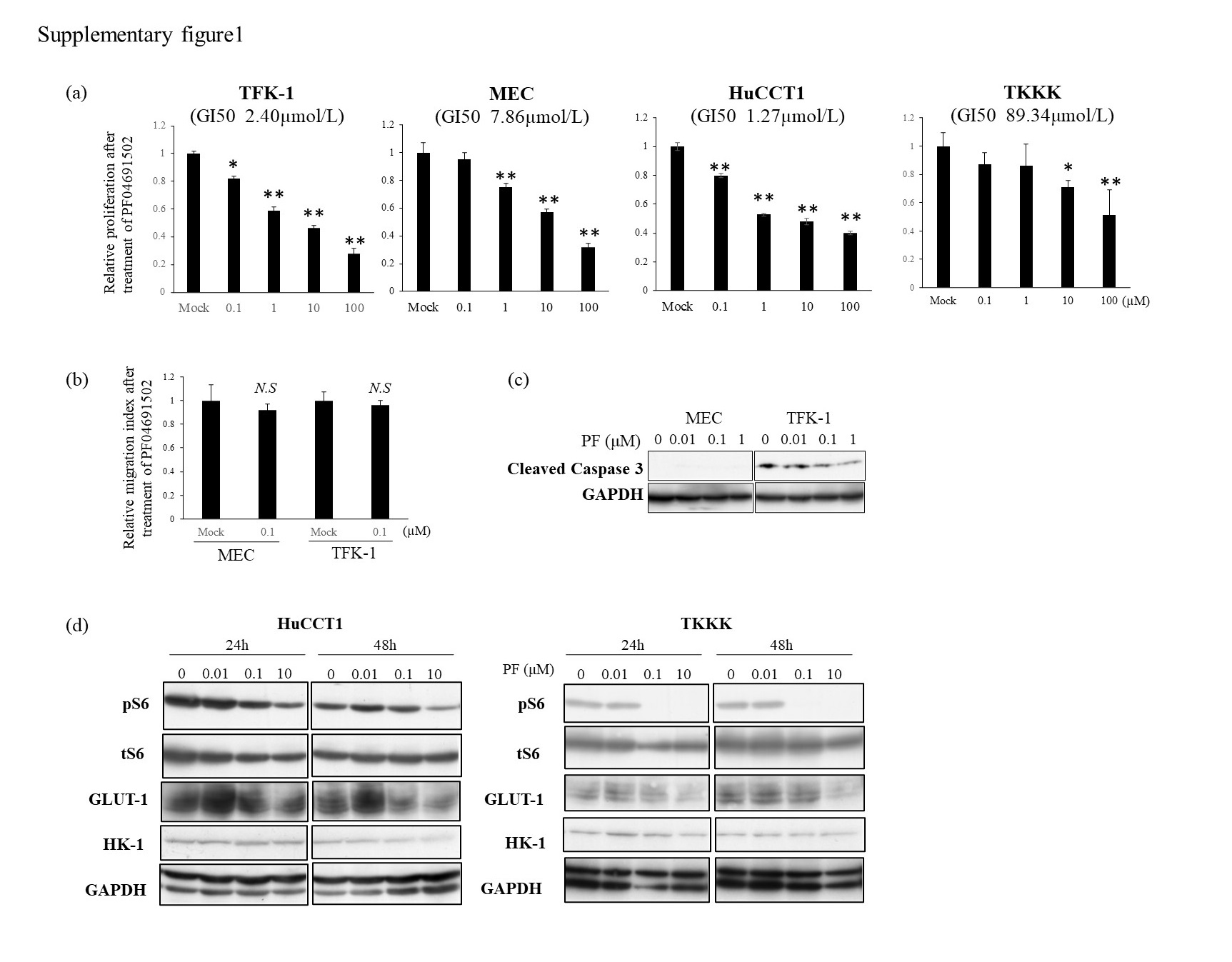

Supplement: Supplementary file 1 — Additional file 1: Supplementary Fig. 1.The inhibitory effect of PF-04691502 treatment on cell proliferation in four CCA cell lines.The inhibitory effect of PF-04691502treatment on migration ability in TFK-1 and MEC cells.Western blotting to investigate the level of cleaved caspase3 under PF-04691502 treatment in TFK-1 and MEC cells. * vs control, P < 0.05, ** vs control, P < 0.001. [file 12876_2023_2815_MOESM1_ESM.jpg]

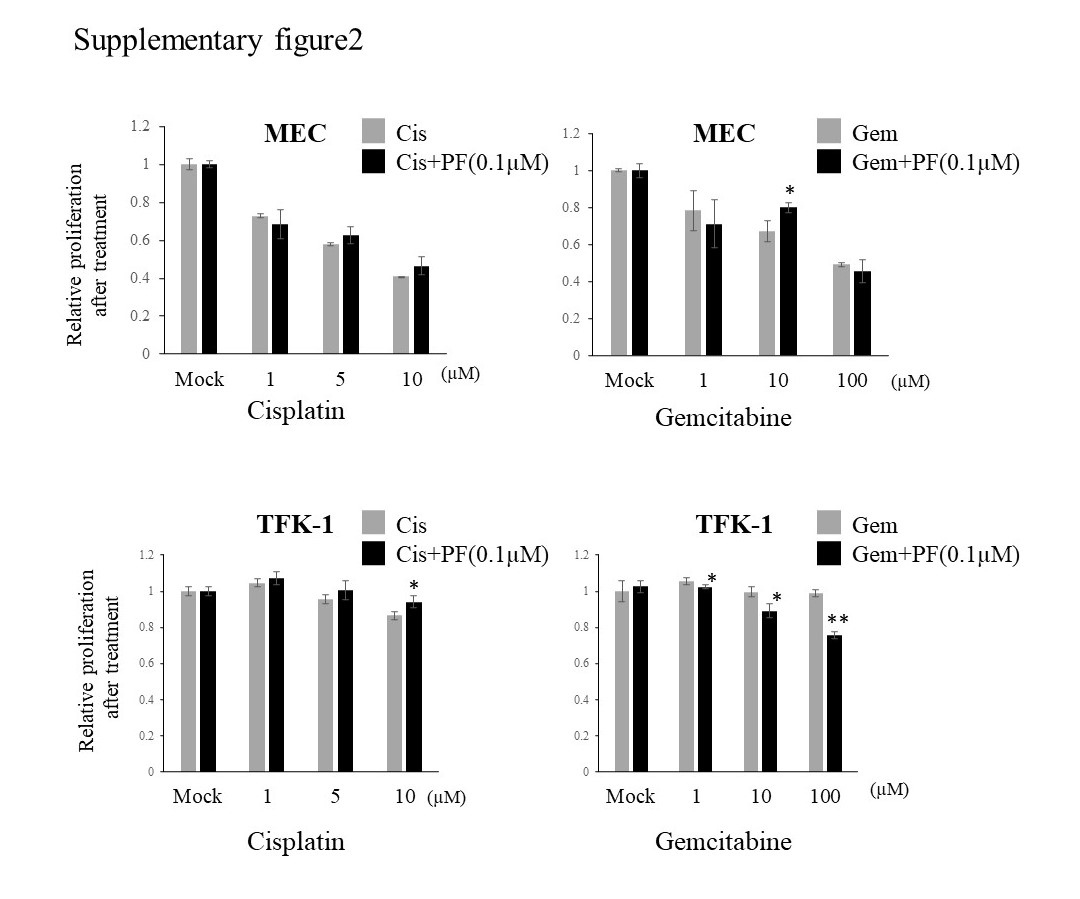

Supplement: Supplementary file 2 — Additional file 2: Supplementary Fig. 2. The inhibitory effect of cisplatin/gemcitabinecombined with PF-04691502in TFK-1 and MEC cells. * vs control, P < 0.05, ** vs control, P < 0.001. [file 12876_2023_2815_MOESM2_ESM.jpg]
