# Supplementary material for: Glucose metabolic upregulation via phosphorylation of S6 ribosomal protein affects tumor progression in distal cholangiocarcinoma
Source: BMC Gastroenterol. 2023 May 16;23:157. doi: 10.1186/s12876-023-02815-2 (PMC10190040; doi:10.1186/s12876-023-02815-2)
Supplement: Supplementary file 3 — Additional file 3. [file 12876_2023_2815_MOESM3_ESM.docx]

Figure s1















Figure s2


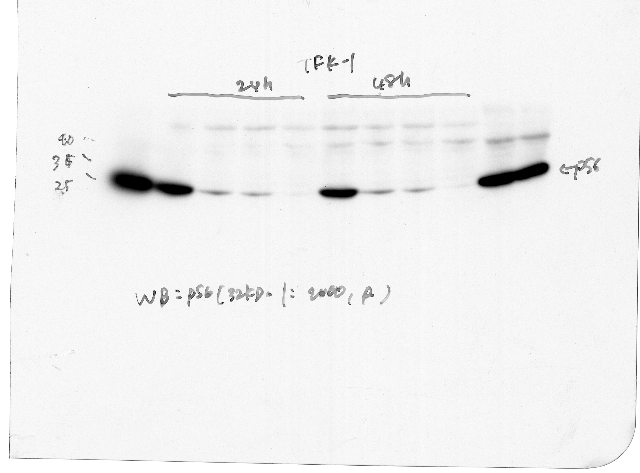

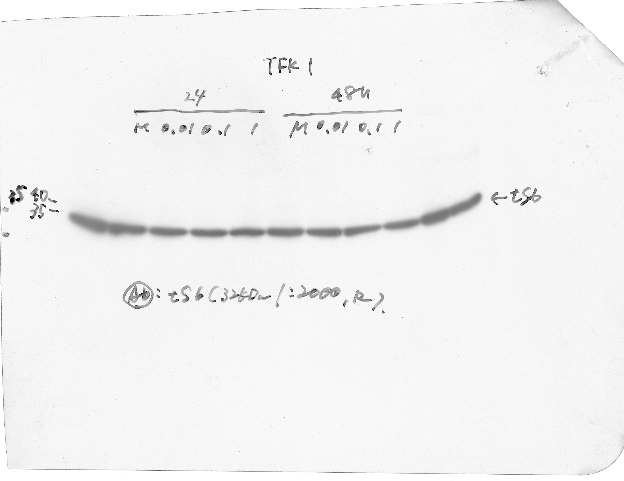


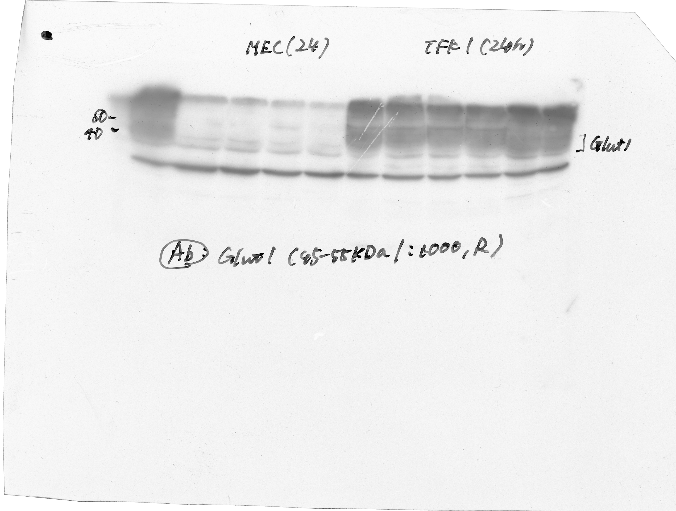

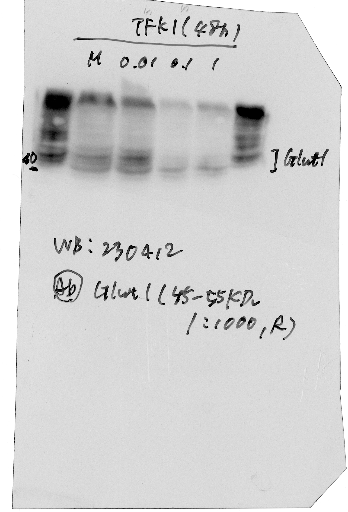


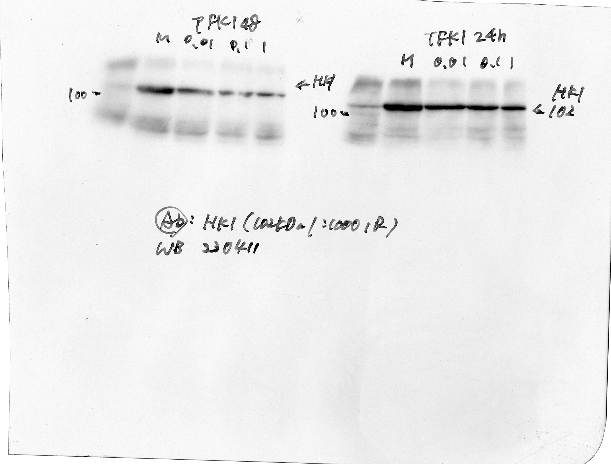

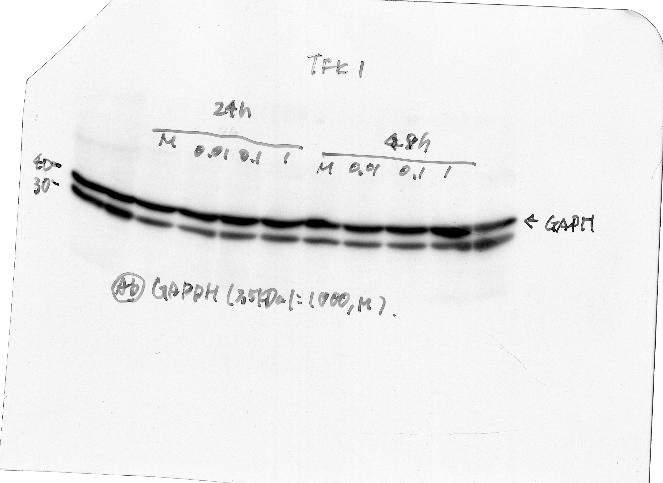


Figure s3


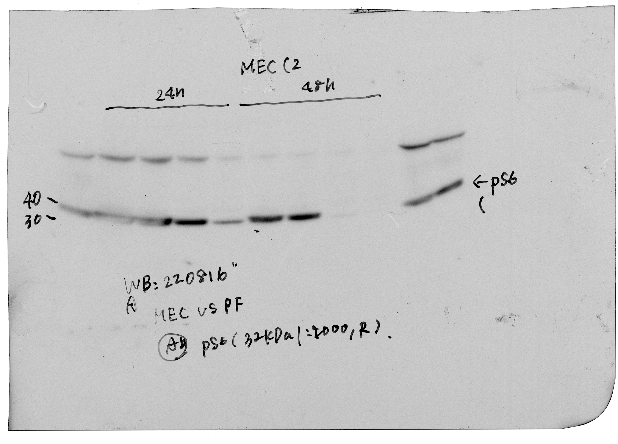

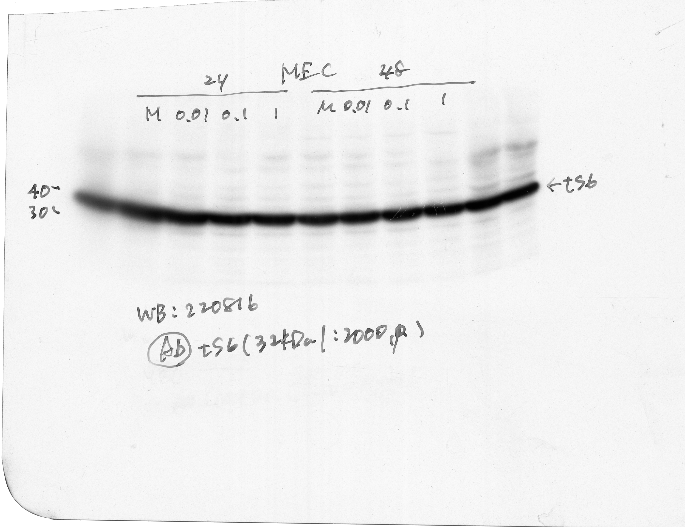


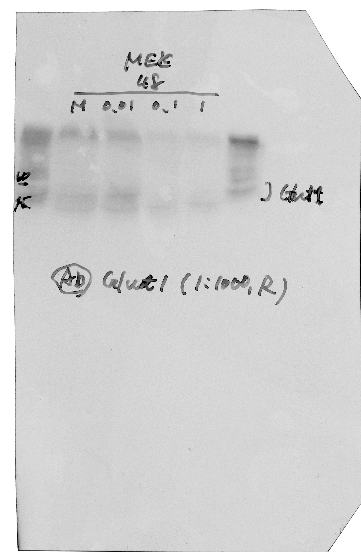

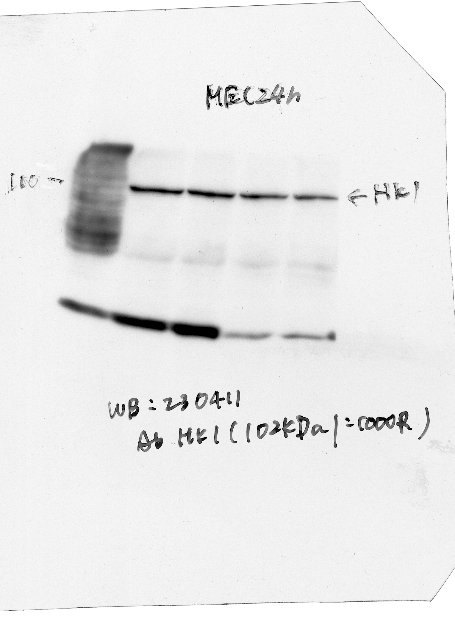

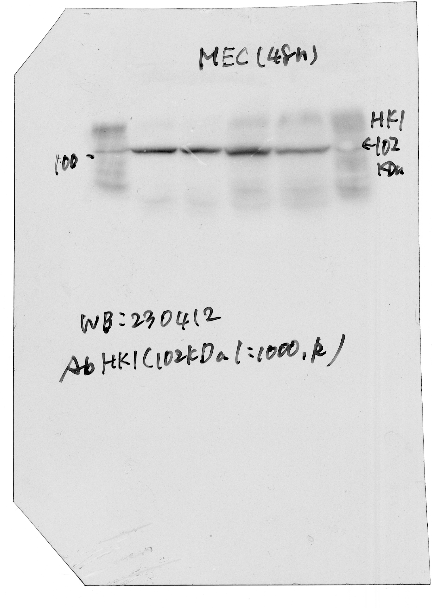


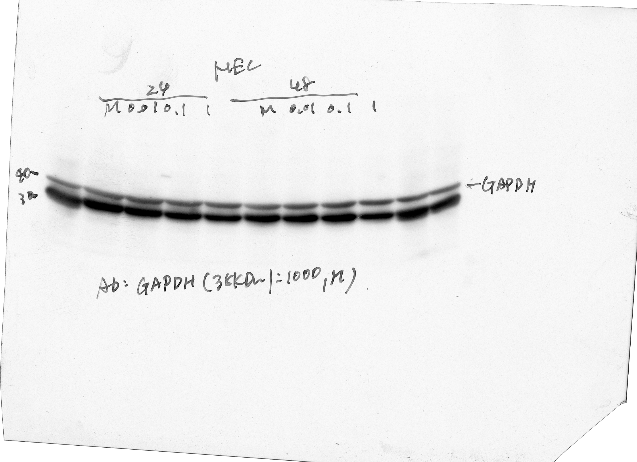


Figure s4


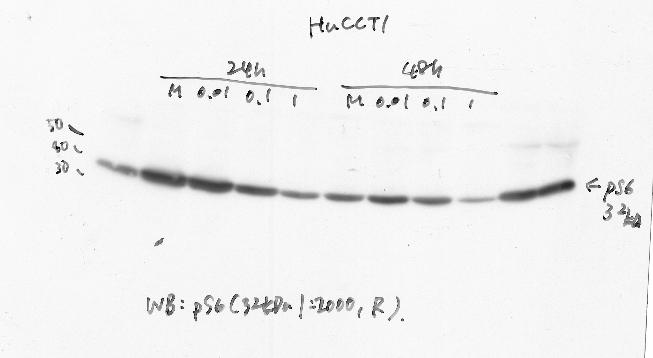

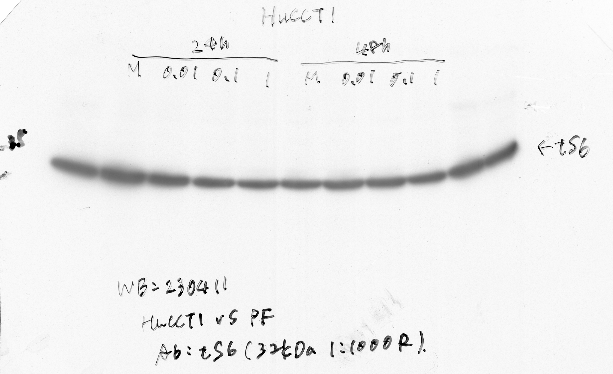


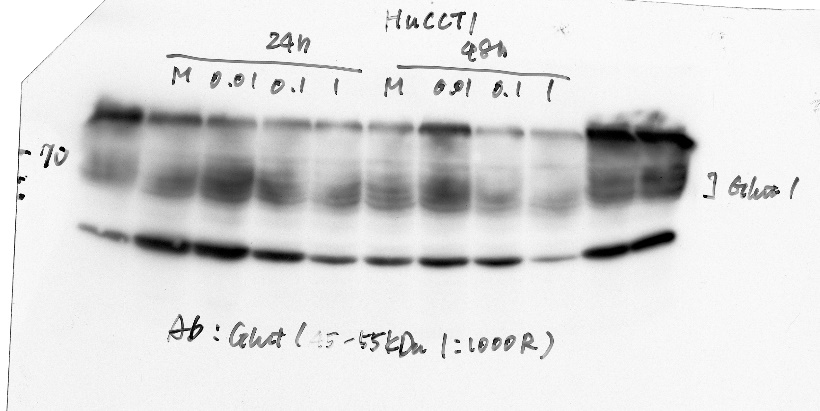

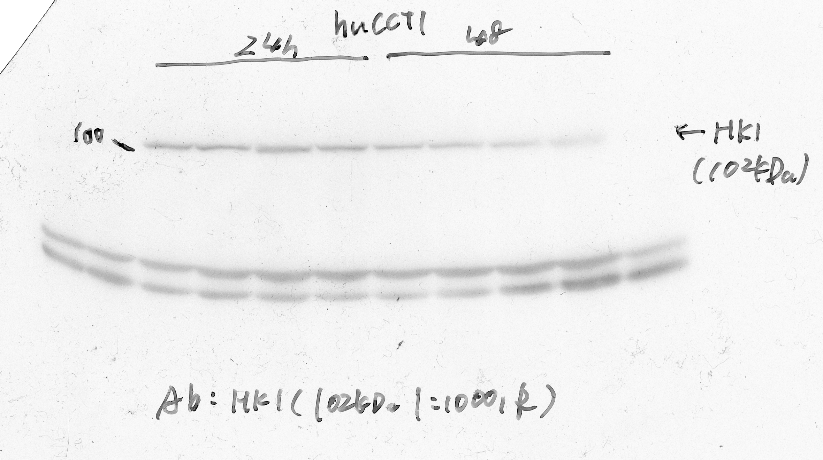


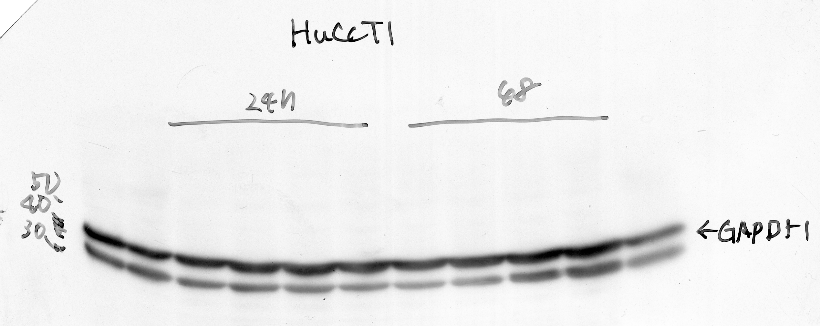


Figure s5


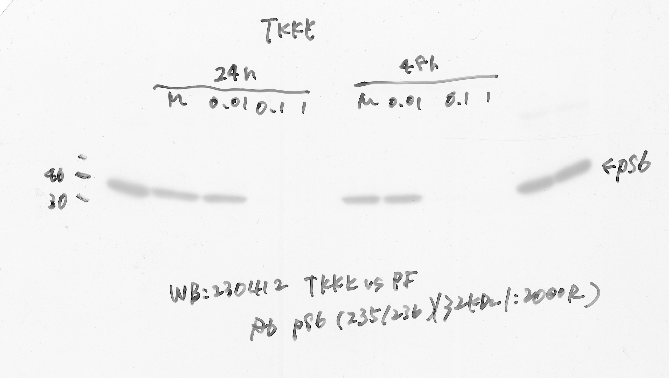

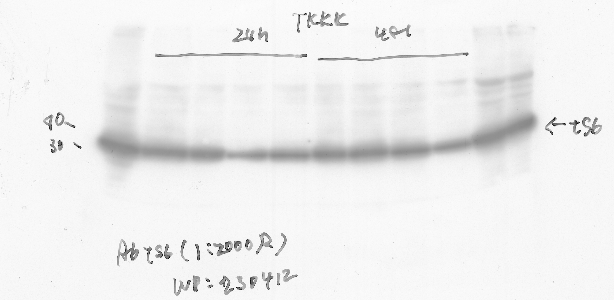


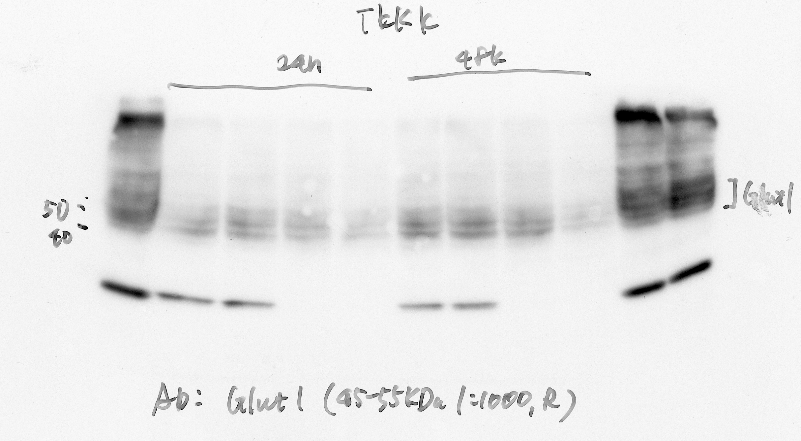

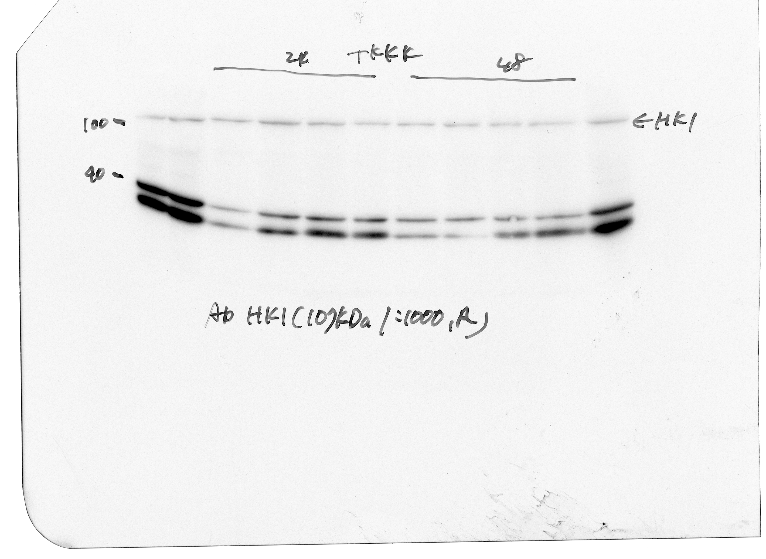


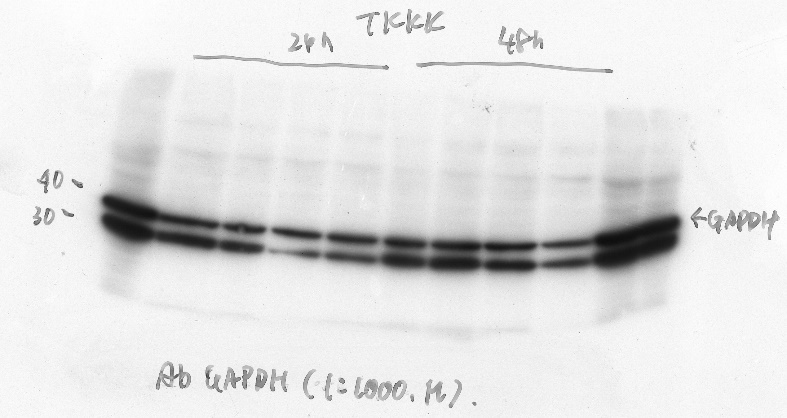


Figure s6


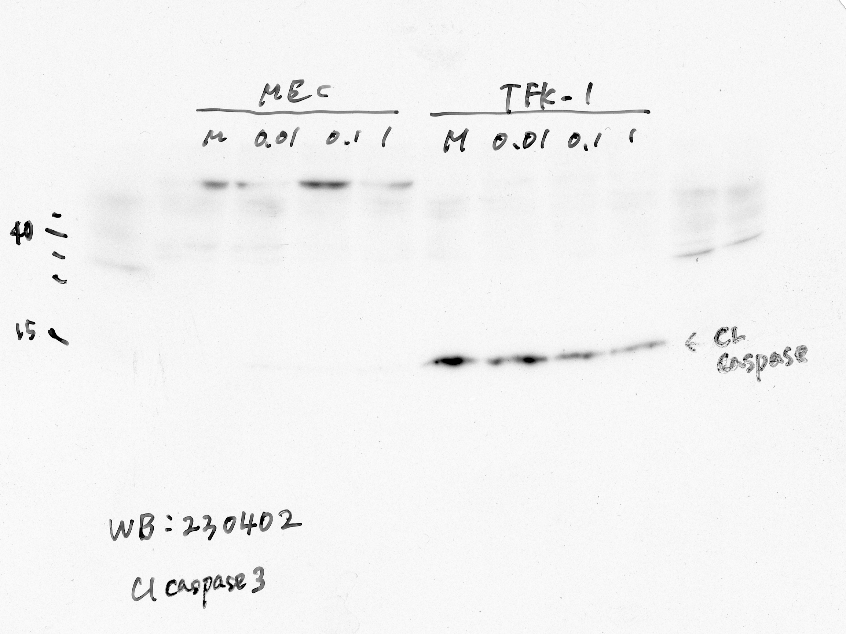


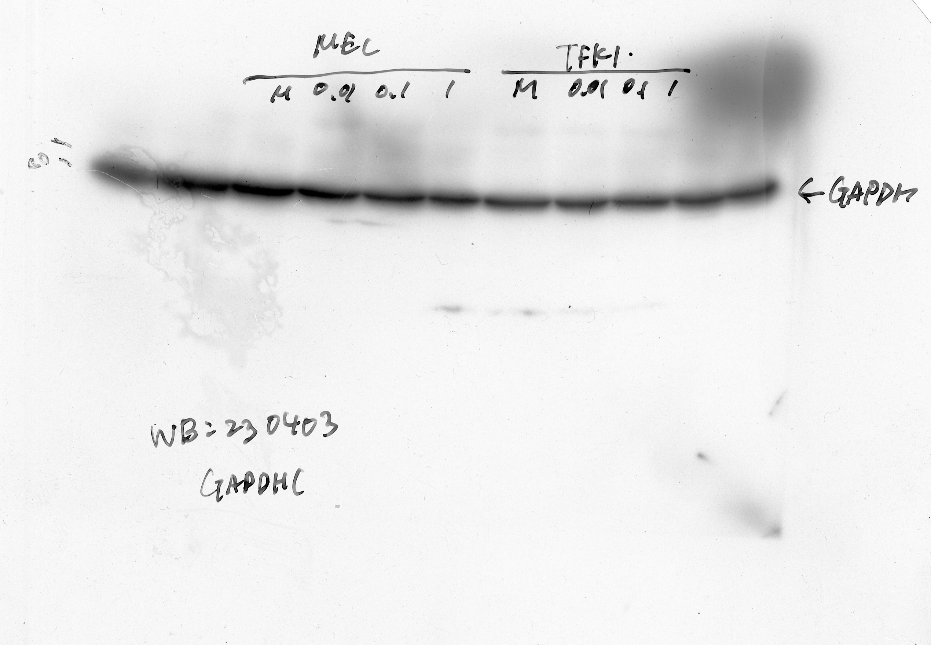


Figure Legends

Figure s1

Western blotting to investigate the level of pS6, tS6, GLUT1, HK-1, and GAPDH in 4 cholangiocarcinoma cell lines.

Figure s2

Western blotting to investigate the effect of pS6, tS6, GLUT1, HK-1, and GAPDH under PF-04691502 treatment in TFK-1 cell line.

Figure s3

Western blotting to investigate the effect of pS6, tS6, GLUT1, HK-1, and GAPDH under PF-04691502 treatment in MEC cell line.

Figure s4

Western blotting to investigate the effect of pS6, tS6, GLUT1, HK-1, and GAPDH under PF-04691502 treatment in HuCCT1 cell line.

Figure s5

Western blotting to investigate the effect of pS6, tS6, GLUT1, HK-1, and GAPDH under PF-04691502 treatment in TKKK cell line.

Figure s6

Western blotting to investigate the effect of cleaved caspase3 and GAPDH under PF-04691502 treatment in TFK-1 and MEC cell lines.
